# “Top-down” Does Not Mean “Voluntary”

**DOI:** 10.5334/joc.28

**Published:** 2018-05-14

**Authors:** Nicholas Gaspelin, Steven J. Luck

**Affiliations:** 1 Binghamton University, State University of New York, US; 2 University of California, Davis, US

**Keywords:** attentional capture, visual attention, selection history

## Abstract

Attention researchers have long debated the roles of top-down and bottom-up mechanisms in controlling attention. Theeuwes (2018) has argued that that top-down control is much less common than typically assumed and that a third mechanism—selection history—plays an underappreciated role in guiding visual attention. Although Theeuwes has made a strong case for the importance of selection history, his arguments for a limited role of top-down mechanisms involve conflating the terms “top-down” and “voluntary.” Cognitive psychologists typically use the term “top-down” processing to refer to any perceptual phenomenon that is influenced by context, learning, or expectation, which would include selection history. This highlights a broad problem in attention capture research: The terms used to describe attentional control are often poorly defined, and much current debate seems to be related to the meaning of words. To move forward in understanding the actual mechanisms of attentional control, we must agree on what terms such as “top-down” and “bottom-up” actually mean.

Traditionally, mechanisms of attentional control have been divided into two distinct classes: top-down and bottom-up. Theeuwes (2018) argues for the inclusion of a third mechanism that may guide attentional allocation: *selection history*. According to this account, implicit mechanisms such as priming or reward history may explain a large amount of the guidance of visual attention. We agree with many of the key points of this review, especially the likelihood that much of attentional guidance is involuntary and unconscious. However, we disagree with the way that Theeuwes has conflated the terms “top-down” and “voluntary.” In this commentary, we will demonstrate that these terms have traditionally meant very different things in the literature on perception and cognition, and we will argue that conflating them leads to confusion rather than clarity.

## Why Terminology Matters

It might seem petty for us to focus on the definition of one little word. After all, shouldn’t we be focusing on the data? However, standardized terminology is crucial for scientific progress. Consider a hypothetical debate about whether the sky is blue. One group of researchers defines “blue” as one color (which would look “light blue” to most of us). They conclude the sky is blue. However, another group of researchers defines “blue” as a different color (which would look “dark blue” to most of us). They conclude the sky is not blue. These two theoretical camps will be doomed to debate forever – or at least until they can agree upon a common definition of the word “blue.” Perhaps the most depressing part of this parable is that both camps agree on the actual spectral content of the sky but believe they disagree because they are using different terms for the same color. Most of us would agree that this hypothetical debate is silly. But it illustrates a key problem. If we cannot agree upon the meanings of “top-down” and “bottom-up,” there is little hope for reaching consensus about the mechanisms of attentional control.

## “Top-down” versus “voluntary”

Theeuwes (2018) defines “top-down” as being equivalent to “voluntary” or “intentional.” He states that “top-down attention is relatively slow” (p. 18), that “top-down selection is controlled” (p. 18) rather than automatic, and that “top-down selection requires effort to shift attention” (p. 19). He also provides several quotes from attention researchers showing that they often stress the voluntary aspects of top-down attentional control. However, close inspection of these quotes reveals that several of the researchers considered “voluntary” attentional control to be a subset of “top-down” attentional control and not equivalent to “top-down.” For example, the statement that “voluntary orienting can be considered aspects of top-down attentional control” (Hopfinger, Buonocore, & Mangun, 2000, p. 284), implies that there are also *involuntary* forms of top-down control (because otherwise the modifier would be unnecessary). Similarly, Baluch and Itti (2011) are cited because they used the phrase “volitional top-down process.” But the glossary of that paper clearly distinguishes between volitional top-down processes and top-down processes more generally. A “top-down process” is defined by Baluch and Itti as “…an automatic, percept-modifying [top-down] mechanism that is pervasive and that volition cannot completely eliminate.” (p. 210). Similarly, a classic study of contextual cuing (Chun & Jiang, 1999) is titled, “Top-down attentional guidance based on implicit learning of visual covariation,” which makes a clear distinction between top-down control and explicit, voluntary control. Thus, “top-down” is not equivalent to “voluntary” in common usage.

So, what does “top-down” mean? Traditionally, “top-down perception” refers to situations in which context, learning, or expectation alters a perceptual process. For example, a standard introductory psychology textbook provides the following definition: “Top-down processing is how knowledge, expectations, or past experiences shape the interpretation of sensory information” (Gazzaniga, Heatherton, & Halpern, 2016, p. 173). Another introductory psychology textbook defines top-down as: “Cognitive (usually perceptual) process directed by expectations (derived from context, past, learning, or both) to form a larger percept, concept, or interpretation” (Galotti, 2014, p. 423). This is echoed in the scientific literature by Baluch and Itti (2011), who define “top-down influence” as “influence on the nervous system due to extra-retinal effects such as intrinsic expectations, knowledge and goals.” All of these definitions say nothing about awareness or intentionality, but do include past experiences (such as selection history).

**Figure 1:**
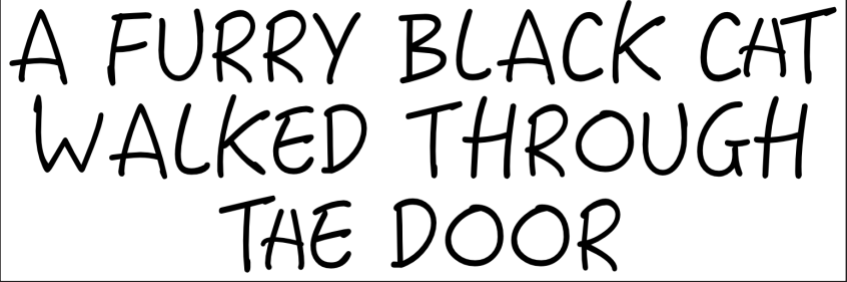
Illustration of a top-down perceptual phenomenon that does not seem voluntary or intentional (adapted from Selfridge, 1955). The “a” in “cat” and the “h” in the “the” have the same letter shape. However, people effortlessly see the words “CAT” and “THE” rather than “CHT” and “TAE”.

A common textbook illustration of top-down perception is shown in Figure 1. The “A” in “CAT” and the “H” in “THE” are exactly the same shape, but top-down knowledge of common English words allows experienced readers to read the words as “CAT” and “THE” without even realizing that they could be seen as “CHT” and “TAE”. Few would assert that this classic example of top-down processing is voluntary (slow, controlled, and effortful). Another common example of top-down perception is the auditory *phonemic restoration effect*, in which a masked sound within a speech signal is restored for familiar but not unfamiliar words (Samuel, 1981). No one would argue that phonemic restoration is voluntary.

Using “top-down” to mean “voluntary” is just not the correct way to use these terms. But, we can understand why the term “top-down” can be confusing. Attention capture researchers often use imprecise language, and we ourselves have been certainly guilty of this. For example, we frequently used the phrase “goal-driven theory” when referring to theories in which intentions or expectations can *indirectly* lead to the capture of attention by stimuli that shared features with the target (Gaspelin, Leonard, & Luck, 2015, 2017, Gaspelin & Luck, in press, 2018). We did not mean to imply that the observers had the goal of focusing attention on these stimuli. Instead, we assumed that the explicit task goal triggered a cascade of events that led ultimately to the capture stimuli that matched the features of the target but were entirely task irrelevant (see Folk, Remington, & Johnston, 1992, for a particularly clear formulation). However, we never made this logic explicit, and it is easy to understand that the term *goal-driven* could be taken to imply direct, voluntary control of attention. And herein lies the problem – common terminology in this area can easily be misinterpreted.

It is worth mentioning that many researchers advocating for selection history have avoided conflating top-down processing with volition. Most notably, a recent review by Awh, Belopolsky, and Theeuwes (2012) defined top-down control as “attentional control that is driven by factors that are ‘internal’ to the observer” (p. 437). This effectively defines top-down as everything that is not bottom-up, forming a clean dichotomy that is consistent with historical usage of the term “top-down.” That article made a compelling argument that the top-down/bottom-up dichotomy has been problematic as a research strategy, because some forms of top-down control are voluntary, slow, and controlled, whereas others are largely involuntary, fast, and automatic. However, this implies that the field should undergo a shift in research strategy, not change the definition of “top-down.”

## Conclusion

We agree with Theeuwes (2018) that selection history is an important avenue for future research and that it shares many key properties with bottom-up control. However, selection history is clearly a form of “top-down” attentional control as this term has historically been used, and redefining “top-down” to mean “voluntary” would be inconsistent with the rest of the field and inevitably lead to confusion rather than progress. On the other hand, “top-down” theories of attention capture have often focused on forms of top-down guidance that are very different from selection history. For example, the contingent involuntary orienting hypothesis (Folk et al., 1992) proposes that participants form a voluntary attentional set that sometimes leads to capture of attention by irrelevant items, and this is very different from something like automatic priming from previous trials. Similarly, the concepts of “singleton detection mode” and “feature search mode” (Bacon & Egeth, 1994) seem very much like voluntary strategies rather than unconsciously learned effects of selection history. As Theeuwes (2018) points out, disentangling conscious top-down strategies from selection history will require moving away from paradigms in which participants perform the same task for a long block of trials and instead using trial-by-trial cuing paradigms. Such paradigms, when used with well-defined terminology, will allow the field to move forward in understanding the roles of volition, awareness, automaticity, selection history, bottom-up processes, and top-down processes in the guidance of attention.

## Author Information

This study was made possible by NIH Grants R01MH076226 and R01MH065034 to S.J.L.
